# Individual differences in the habitual use of cognitive reappraisal predict the reward-related processing

**DOI:** 10.3389/fpsyg.2015.01256

**Published:** 2015-09-01

**Authors:** Liyang Sai, Sisi Wang, Anne Ward, Yixuan Ku, Biao Sang

**Affiliations:** ^1^The Key Lab of Brain Functional Genomics, MOE & STCSM, School of Psychology and Cognitive Science, East China Normal University, Shanghai, China; ^2^Department of Psychology, Northwestern University, Evanston, IL, USA; ^3^NYU-ECNU Institute of Brain and Cognitive Science, NYU Shanghai and Collaborative Innovation Center for Brain Science, Shanghai, China; ^4^School of Preschool and Special Education, East China Normal University, Shanghai, China

**Keywords:** cognitive reappraisal, reward processing, emotion regulation, feedback negativity, event-related potentials

## Abstract

Recent studies have shown that instructed cognitive reappraisal can regulate the neural processing of reward. However, it is still unclear whether the habitual use of cognitive reappraisal in everyday life is related to brain activity involved in reward processing. In the present study, participants’ neural responses to reward were measured using electroencephalography (EEG) recorded during a gambling task and their tendency to use cognitive reappraisal was assessed using the Emotion Regulation Questionnaire (ERQ). Event-related potential (ERP) results indicated that losses on the gambling task elicited greater negative reward-related feedback negativity (FN) than gains. The differential FN between losses and gains was significantly correlated with cognitive reappraisal scores across participants such that individuals with a higher tendency to use cognitive reappraisal showed stronger reward processing (i.e., amplified FN difference between losses and gains). This correlation remained significant after controlling for expressive suppression scores. However, expressive suppression *per se* was not correlated with FN differences. Taken together, these results suggest that the habitual use of cognitive reappraisal is associated with increased neural processing of reward.

## Introduction

Receiving a reward elicits positive feelings that aid in learning and adaption (Walsh and Anderson, 2012). Conversely, abnormal reward processing underlies a variety of mental disorders including addiction, impulse control disorders, and depression (Koob and Le Moal, 2001; Foti and Hajcak, 2009). It is therefore important to understand how reward processing is regulated.

Cognitive reappraisal, one of the most widely used emotion regulation strategies, seems a promising method for regulating reward processing. Cognitive reappraisal involves construing a potentially emotion-eliciting situation in a way that changes its emotional impact (Lazarus and Alfert, 1964). It has consistently been found that cognitive reappraisal can not only reduce self-reported negative emotional experience, peripheral physiology, and brain activity associated with emotion processing, such as the late positive potential (LPP) in event-relate potentials (ERPs; Hajcak et al., 2010) and amygdala activation (Ochsner and Gross, 2008); but also enhance self-reported positive emotional experience and its associated Cardiovascular activity (e.g., heart rate and cardiac output) and LPP (Baur et al., 2015; Demaree et al., 2004; Pavlov et al., 2014).

Delgado et al. (2008) were the first to examine whether cognitive reappraisal could influence brain activity involved in reward processing. In their study, participants were instructed to either respond normally to a colored square that predicted a potential monetary reward or to down-regulate their emotional responses to the square. They reported decreased neural activity in striatum while participants were engaged in reward regulation, which represented reward anticipation (Delgado et al., 2008). Cognitive reappraisal has also been shown to down-regulate brain activity in the ventral striatum during the outcome period after reward signals are presented (Staudinger et al., 2009; Yang et al., 2013). Furthermore, Langeslag and van Strien (2013) expanded upon Delgado et al. (2008) using a similar task and demonstrated that cognitive reappraisal can also enhance emotional responses to stimuli that predict reward when participants were asked to up-regulate their response to reward-related stimuli (Langeslag and van Strien, 2013). Taken together, these findings suggest that cognitive reappraisal changes brain activity associated with reward processing.

In the aforementioned studies’ experimental designs, participants were instructed to use cognitive reappraisal to regulate their responses to the reward-related stimuli. However, even without explicit instructions, people report using cognitive reappraisal frequently in everyday life (Gross et al., 2006). This habitual use of cognitive reappraisal might have an accumulated influence on reward processing. Thus, the present study was about to investigate the relationship between the habitual use of cognitive reappraisal and reward processing. An individual-difference based approach was typically used to investigate the relationship between habitual cognitive reappraisal (gaged by self-report) and emotion-related brain activity across individuals, and the increased use of cognitive reappraisal in everyday life was found associated with reduced amygdala activity and augmented prefrontal activity in response to negative stimuli (Drabant et al., 2009; Vanderhasselt et al., 2013).

Thus, the present study adopted the individual-difference approach to examine the relationship between individual differences in the habitual use of cognitive reappraisal and the neural responses to reward during a gambling task. Specifically, we recorded EEG from individuals while they performed a gambling task. This paradigm allowed us to measure their neural responses to gains and losses. We used feedback negativity (FN) to assess participants’ reward processing. FN typically peaks around 300 ms after feedback presentation and has a greater negative slope following negative outcomes than positive outcomes (Gehring and Willoughby, 2002). Traditional reinforcement learning theory of FN interpreted this as a reflection of whether outcomes were better or worse than expected (Holroyd and Coles, 2002). However, recent evidence suggests that FN represents neural activities associated with reward processing (Carlson et al., 2011; Foti et al., 2011; Bress and Hajcak, 2013). Particularly, the difference in FN between loss and gain trials has been shown to be significantly correlated with activity in the mesocorticolimbic reward circuit, including ventral striatum and medial prefrontal cortex (mPFC; Carlson et al., 2011), as well as self-reported reward responsiveness (Bress and Hajcak, 2013). The habitual use of cognitive reappraisal was measured using the Emotion Regulation Questionnaire (ERQ), which has been widely used in previous studies (Gross and John, 2003; Drabant et al., 2009; Vanderhasselt et al., 2013).

Previous studies have shown that instructed cognitive reappraisal can both up-regulate and down-regulate brain activities associated with reward processing (e.g., Delgado et al., 2008; Langeslag and van Strien, 2013). In a similar way, the habitual use of cognitive reappraisal may relate to enhanced or reduced neural responses to reward. Thus, we hypothesize that the habitual use of cognitive reappraisal will be significantly correlated with reward processing, which would be reflected in the differential FN amplitude between losses and gains (Drabant et al., 2009). In addition, some previous findings suggest that there is gender difference on both reward processing and cognitive reappraisal (e.g., Kamarajan et al., 2008; Domes et al., 2010), thus, we also include gender as a factor in the present study.

## Materials and Methods

### Participants

Twenty-one participants (11 females, Mean age = 19.90 years, SD = 2.17 years) were recruited from East China Normal University and compensated for their participation. One female was excluded from further analysis because of excessive EEG artifacts. All participants had normal or corrected-to-normal vision and were right-handed. The study was approved by the Ethics Committee of East China Normal University.

### Gambling Task

In order to elicit reward-related brain activity, we employed a gambling task (Foti and Hajcak, 2009). During each trial, an image with two adjacent doors was presented and participants were asked to select one by pressing either a left or a right button. After participants selected a door, a feedback appeared (“+4,” “–2”) and indicated whether they had won ¥4 (about 0.7 dollars) or lost ¥2 (about 0.35 dollars).

Participants were seated one meter away from a 17-inch computer monitor. Stimuli were presented in black font on a white background. Each trial began with a fixation cross (500 ms), followed by the image of two doors. The doors remained on the screen until the participant made a button response. Once a door was chosen, another fixation was presented for 3000 ms, followed by feedback (2000 ms) for the choice. After the feedback, another fixation was presented for 1000 ms. Participants pressed the space bar to start the next trial. Unbeknownst to participants, feedback in each trial was bogus and was administered randomly (wining ¥4 or losing ¥2). There were 40 loss trials and 40 gain trials. All participants were paid 30 RMB (about 5 dollars) after the experiment.

### EEG Acquisition

Electroencephalography data were recorded with 32 Ag/AgCl electrodes (international 10–20 arrangement) embedded in an elastic cap and were amplified by SynAmps 2 (Neuroscan Inc., USA). Online recordings were referenced to the left mastoid and data were then re-referenced offline to the mean of both mastoids. Electrode impedances were kept below 5 kΩ. Vertical electro-oculograms (EOGs) were recorded above and below the right eye and horizontal EOGs were recorded from electrodes placed at the outer canthus of both eyes. The EEG and EOG were amplified from DC to 100 Hz and digitized online with a sampling rate of 500 Hz. For offline analyses, continuous EEGs were first filtered with a low-pass filter (30 Hz cut-off, 24 dB/ct). Continuous EEGs were then segmented into epochs from –200 ms to 1000 ms, with 0 ms locked to the feedback stimuli. The artifact correction procedure provided by Scan 4.3 (Neuroscan) was used to remove blink artifacts, and trials with artifacts exceeding ± 100 μV were excluded from averaging. FN was calculated using the difference between loss and gain trials (loss minus gain) and was averaged between 280 and 380 ms after feedback onset (Bress and Hajcak, 2013).

### Cognitive Reappraisal Scores

The ERQ was administered to measure the habitual use of cognitive reappraisal. This scale consists of 10 items, six of which assess individual differences in cognitive reappraisal (e.g., “I control my emotions by changing the way I think about the situation I’m in”). The remaining four items gage individual differences in expressive suppression use (e.g., “I control my emotions by not expressing them”). Ratings were made on a 1 (strongly disagree) to 7 (strongly agree) scale. The six cognitive reappraisal items were summed to calculate cognitive reappraisal scores and the four suppression items were totaled to determine expressive suppression scores (Gross and John, 2003).

### Statistical Analyses

A two-way repeated measure analysis of variance (ANOVA) on the difference FN was performed with electrode site (Fz, FCz, Cz, CPz, and Pz) as a within-subjects variable and gender (male, female) as a between-subjects variable. Pearson’s correlation was calculated to establish the strength of the relationship between ERPs and ERQ scores. All statistical analyses were conducted with SPSS 20.0 (IBM Inc.). The Greenhouse-Geisser correction was applied when the assumption of sphericity was violated. *Post hoc* comparisons were computed with Fisher’s Protected Least Significant Difference (LSD).

## Results

### Cognitive Reappraisal Scores

The mean reappraisal score was 31.40 (SD = 5.58) with a range from 21 to 41, and the mean suppression score was 16.00 (SD = 4.65), and ranged from 4 to 24.

### ERP Results

**FIGURE 1:**
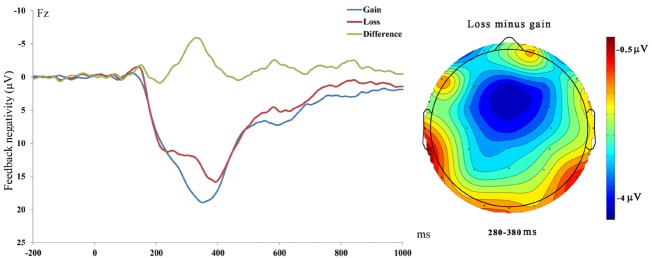
**Left: Feedback-locked ERPs at Fz for loss, gain and loss-gain difference trials.** Right: Scalp. Distribution of the difference between loss and gain trials from 280 to 380 ms.

**FIGURE 2:**
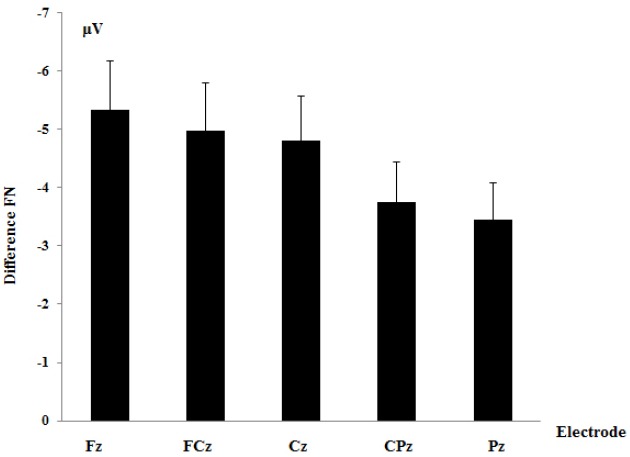
**Difference FN amplitudes between losses and gains in all five electrodes**.

A two-way repeated measure analysis of variance (ANOVA) on the difference FN was performed with electrode site (Fz, FCz, Cz, CPz, and Pz) as a within-subjects variable and gender (male, female) as a between-subjects variable. The main effect of electrodes was significant, *F* (1, 19) = 4.43, *p* = 0.03, ${\text{η}}_{\text{p}}^{\text{2}}$ = 0.20. Planned contrast showed that difference FN at Fz (Mean ± SE = –5.34 ± 3.70 μV) was larger than the other four electrodes (Mean ± SE = –4.25 ± 2.96 μV), *p* = 0.047. The main effect of gender, *F* (1, 19) = 3.10, *p* = 0.095, ${\text{η}}_{\text{p}}^{\text{2}}$ = 0.15, and the interaction between electrodes and gender, *F* (1, 19) = 0.25, *p* = 0.91, ${\text{η}}_{\text{p}}^{\text{2}}$ = 0.01 were not significant (see Figures 1 and 2).

### Neural-behavioral Correlation

**FIGURE 3:**
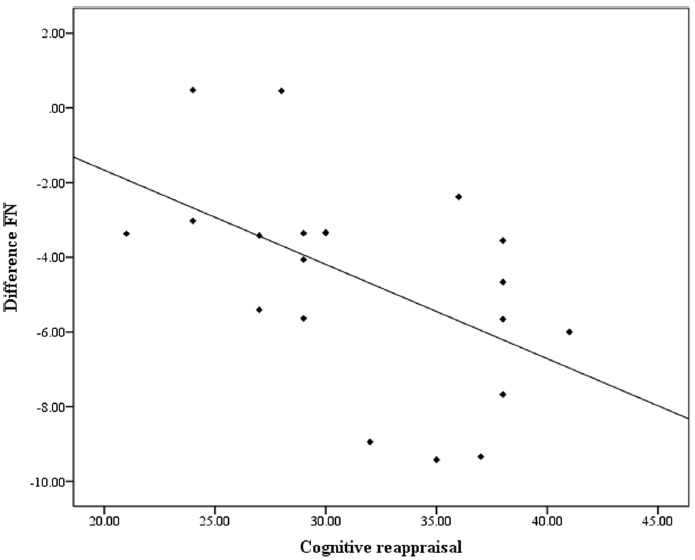
**The correlation between cognitive reappraisal scores and difference FN**.

As shown in the results above, FN was most prominent at Fz and the FN amplitude at Fz was therefore used for the correlation analysis. There was a significant negative correlation between the differential FN and cognitive reappraisal scores (*r* = –0.52, *p* = 0.019). The correlation remained significant (*r* = –0.50, *p* = 0.028) after conducting a partial correlation to control for expressive suppression scores. However, cognitive reappraisal scores were not correlated with FN amplitude in either the gain (*r* = 0.02, *p* = 0.84) or the loss (*r* = –0.18, *p* = 0.54) conditions (see Figure 3). Another Pearson’s correlation was conducted between expressive suppression scores and differential FNs and did not reach significance (*r* = –0.15, *p* = 0.52). Suppression scores were also not correlated with loss (*r* = 0.31, *p* = 0.13) or gain (*r* = 0.35, *p* = 0.18) FN amplitudes.

## Discussion

A number of recent studies have demonstrated that instructed cognitive reappraisal can both down-regulate and up-regulate neural processing of reward (e.g., Delgado et al., 2008; Staudinger et al., 2009, 2011; Langeslag and van Strien, 2013). In the current study, we investigated whether individual differences in everyday reappraisal use also correlates with neural reward processing. The present study employed a gambling task and the ERQ to address this question.

Consistent with previous studies (Holroyd et al., 2004; Hajcak et al., 2007), we found that FN was more negatively deflected following losses than gains and was most prominent at frontal sites. These data suggest that our task elicited a reliable reward-related FN. Several recent studies have found that only the differential FN between gains and losses (neither FN for gains or FN for losses) was correlated with activity in the mesocorticolimbic reward circuit, including ventral striatum and medial prefrontal cortex (mPFC; e.g., Carlson et al., 2011), as well as self-reported reward responsiveness (Bress and Hajcak, 2013). These findings suggest that the differential FN between gains and losses indexed reward processing. The differential FN (losses minus gains) was then used to assess the neural processing of reward (Carlson et al., 2011; Bress and Hajcak, 2013). We did not find the gender effect on differential FN. However, it should be noted that the P value for gender was marginal (*p* = 0.095), which might be due to the lower power after splitting the group. Future study designed to look at the gender effect with more participants will be of interest.

Consistent with our hypothesis, the differential FN was correlated with cognitive reappraisal scores such that the more frequently a participant used cognitive reappraisal in daily life, the more pronounced their reward processing, which was reflected in their differential reaction to losses compared with gains. This correlation remained significant after controlling for expressive suppression scores, indicating that the enhanced reward processing cannot be attributed to the tendency to use expressive suppression. It has been suggested that the reward system plays a central role in adaptation to the environment (e.g., Holroyd and Coles, 2002; Schultz, 2006). Thus, participants learned to use cognitive reappraisal to adjust their responses to reward processing in daily life. With more frequent use, individuals could initiate cognitive reappraisal more implicitly to up-regulate their responses when the reward-related stimuli were presented (Gyurak et al., 2011). Individuals with more habitual use of cognitive reappraisal showed enhanced neural responses to reward as well in the present study. A few recent studies suggest that instructed cognitive reappraisal could reliably down-regulate and up-regulate brain activity associated with reward (e.g., in the ventral striatum, e.g., Staudinger et al., 2009; Langeslag and van Strien, 2013). Our results expand upon these findings by demonstrating that the habitual use of cognitive reappraisal in everyday life is related to increased brain activity associated with reward processing.

Previous ERP studies on cognitive reappraisal have focused mainly on the modulation of LPP amplitude, which was found to be modulated by emotional stimuli. Larger LPP amplitude was related to increased arousal (for a review, see Hajcak et al., 2010). The LPP amplitude was further found reduced/enhanced when participants were asked to use cognitive reappraisal to down-regulate/up-regulate feelings elicited by emotional images (e.g., Krompinger et al., 2008; Moser et al., 2009; Thiruchselvam et al., 2011). Our results indicate that cognitive reappraisal could also modulate reward-related brain activity.

Our novel findings also have important clinical implications. Evidence suggests that reward dysregulation underlies a variety of mental disorders, including depression, anxiety, and bipolar disorder (Koob and Le Moal, 2001). Specifically, research has found that decreased FN amplitude in response to reward is associated with depression (Foti and Hajcak, 2009) and anxiety (Gu et al., 2010). The results of the present study provide evidence linking stronger FN difference between losses and gains with the habitual use of cognitive reappraisal, and suggest that less frequent use of cognitive reappraisal in everyday life is correlated with blunted response to reward, which may be an important contributor to the development of depression and anxiety. Further work should test this hypothesis by investigating the relationship among habitual use of cognitive reappraisal, reward processing, and depression (or anxiety).

### Conflict of Interest Statement

The authors declare that the research was conducted in the absence of any commercial or financial relationships that could be construed as a potential conflict of interest.
